# The carcinogenicity of polycyclic hydrocarbon epoxides in newborn mice.

**DOI:** 10.1038/bjc.1975.24

**Published:** 1975-02

**Authors:** P. L. Grover, P. Sims, B. C. Mitchley, F. J. Roe

## Abstract

Benz(a)anthracene injected subcutaneously during the first 3 days of life caused a dose related increase in the incidence of liver and lung tumours in Swiss mice but over a similar dose range, the K region epoxide of benz(a)anthracene was less effective. Neonatally injected 7-methylbenz(a) was considerably more active than its K region epoxide in increasing the incidence of liver tumours in males. Both the parent compound and the epoxide slightly raised the incidence of lung tumours. Both chrysene and its K region epoxide increased liver tumour incidence but not lung tumour incidence. The K region epoxides of dibenz(a,h)-anthracene and 3-methylcholanthrene were without apparent effect on the incidence of liver, lung or other tumours despite indications from previously reported studies that the parent hydrocarbons are active at the same dose levels. The K region epoxide of phenanthrene had no effect on the incidence of any kind of neoplasm.


*Br. J. Cancer* (1975) **31**, 182

### THE CARCINOGENICITY OF POLYCYCLIC HYDROCARBON EPOXIDES IN NEWBORN MICE

P. L. GROVER, P. SIMS, B. C. V. MITCHLEY AND F. J. C. ROE

*From the Chester Beatty Research Institute, Institute of Cancer Research, Royal Cancer Hospital, Fulham Road, London, SW3 6JB*

Received 9 August 1974. Accepted 27 September 1974

**Summary.—Benz(a)anthracene injected subcutaneously during the first 3 days of life caused a dose related increase in the incidence of liver and lung tumours in Swiss mice but over a similar dose range, the K region epoxide of benz(a)anthracene was less effective. Neonatally injected 7-methylbenz(a)anthracene was considerably more active than its K region epoxide in increasing the incidence of liver tumours in males. Both the parent compound and the epoxide slightly raised the incidence of lung tumours. Both chrysene and its K region epoxide increased liver tumour incidence but not lung tumour incidence. The K region epoxides of dibenz(a,h)-anthracene and 3-methylcholanthrene were without apparent effect on the incidence of liver, lung or other tumours despite indications from previously reported studies that the parent hydrocarbons are active at the same dose levels. The K region epoxide of phenanthrene had no effect on the incidence of any kind of neoplasm.**

THE HYPOTHESIS that the carcinogenicity of polycyclic hydrocarbons is associated with the formation of epoxide metabolites (Grover, Hewer and Sims, 1971, 1972; Keysell *et al.*, 1973) is supported by some experimental evidence but remains unproven. The original comparative carcinogenicity data from 3 laboratories showed that K region epoxides were less potent than the parent hydrocarbons either when applied topically or when administered subcutaneously (Boyland and Sims, 1967; Sims, 1967; Miller and Miller, 1967; Van Duuren *et al.*, 1967). More recent studies with epoxides in 2 *in vitro* transformation systems (Berwald and Sachs, 1963; Chen and Heidelberger, 1969) have demonstrated that several K region derivatives are more active than the corresponding hydrocarbons in inducing malignant transformation (Grover *et al.*, 1971; Marquardt *et al.*, 1972; Huberman *et al.*, 1972), but others are not (Marquardt *et al.*, 1974). The epoxides can also act as alkylating agents (Grover and Sims, 1970) and as mutagens (Ames, Sims and Grover, 1972; Huberman *et al.*, 1971; Cookson, Sims and Grover, 1971; Fahmy and Fahmy, 1973). Rapidly dividing cell populations, like those used in the *in vitro* transformation systems, might be expected to be more susceptible to reactive carcinogens, like epoxides, than mitotically inactive tissues if reactions with cellular constituents at a particular stage of the cell cycle are important (Bertram and Heidelberger, 1974; Marquardt, 1974). The newborn mouse, which is susceptible to carcinogenic polycyclic hydrocarbons (Roe, Rowson and Salaman, 1961; Roe, Mitchley and Walters, 1963; Walters and Roe, 1964, 1966) can be considered as a convenient source of several types of rapidly dividing cells. Consequently we have tested the K region epoxides derived from phenanthrene, benz(a)anthracene, 7-methylbenz(a)anthracene, dibenz(a,h)-anthracene, chrysene and 3-methylcholanthrene, together with some of the parent hydrocarbons, for carcinogenicity using newborn mice. This paper presents the results that were obtained in these experiments.

### MATERIALS AND METHODS

The polycyclic hydrocarbon epoxides, phenanthrene 9,10-oxide, benz(a)anthracene 5,6-oxide, 7-methylbenz(a)anthracene 5,6-oxide, dibenz(a,h)anthracene 5,6-oxide, chrysene 5,6-oxide and 3-methylcholanthrene 11,12-oxide were prepared from the parent hydrocarbons (Newman and Blum, 1964; Boyland and Sims, 1965; Sims 1966).

Two experiments were undertaken following similar protocols. For each experiment Swiss mice from a specific pathogen-free colony were time mated so as to produce a large number of litters over the course of 2 or 3 days. Newborn mice from each litter were randomly allocated to various treatment and control groups and 6 or 7 similarly treated baby mice derived from different litters returned to each mother mouse that had contributed her own litter to the pool.

Test compounds were suspended in polyethylene glycol (PEG) 400 and injections of 0·02 ml of the suspension were given by introducing a 25 gauge hypodermic needle through the skin near the root of the tail and threading it subcutaneously to deliver the injected material in the interscapular region. Injections were given to every mouse on the first day or first 3 days of life. The details of treatments and numbers of mice of each sex in each group are shown in Table I (Experiment I) and Table III (Experiment II).

Surviving mice were weaned when they were 21 days old. Thereafter, males and females were separated and housed 6 per cage in metal boxes. A mixture of sawdust and wood shavings was used as bedding. The mice were fed on a standard cubed diet (Formulation 86 from C. Holdman and Sons (Plowco Feeds), Byers Lane, South Godstone, Surrey) and water was given *ad libitum*.

Mice were inspected daily, and at weekly intervals they were weighed and palpated for tumours and other lesions. Mice that were sick or had palpable tumours were killed and examined by a standard post-mortem procedure. The experiment was terminated when surviving mice were between 70 and 75 weeks old. A full routine post-mortem examination, which included distension of the urinary bladder with fixative, was carried out. The number of lesions thought to be neoplasms and the size of the largest such lesion in each organ affected were recorded.

All tissues with suspected neoplasms were fixed in Bouin's solution. Haematoxylin and eosin stained 5 $\mu$m paraffin wax sections were prepared and examined microscopically.

### RESULTS

The results of the two experiments in terms of tumour incidence are summarized in Tables II and IV.

### *Comparison of benz(a)anthracene and its K region epoxide*

Benz(a)anthracene (Groups A, B and C) had a dose related effect on the incidence of liver tumours in male mice and of lung tumours in mice of both sexes. The effect on incidence was manifest in terms both of the proportion of animals killed between the 70th and 75th weeks of the experiment that had at least one tumour of either type and in terms of the proportion of such animals with multiple tumours of these types. In all 3 groups treated with benz(a)anthracene the incidence of both lung and liver tumours was higher than in mice exposed to the vehicle, PEG 400, only (Group G). By comparison, the epoxide of benz(a)-anthracene was less active than the parent compound. At the lowest level of dosage (Group F–3 × 50 $\mu$g) the incidence of the 2 kinds of tumour was not very different from that in Group G. At the 2 higher levels (Groups D and E) treatment was associated with an increase in incidence of liver tumours in males, but the effect was more marked in Group E than in Group D and it was only in Group E females that an increased incidence of lung tumours was apparent.

Histologically, the liver tumours were all of parenchymal cell origin, ranging in appearance from well differentiated

TABLE I.—*Experiment I: Comparison of Effects of Benz(a)anthracene and of its K Region Epoxide*

| Group | Treatment on Days 0, 1 and 2 (all treatments given in 0·02 ml PEG 400): Compound | Dose per treatment | No. of mice injected (♂ + ♀) | No. of mice weaned (Day 21): Total | ♂ | ♀ |
|---|---|---|---|---|---|---|
| A | Benz(a)anthracene | 200 μg | 87 | 38 | 19 | 19 |
| B | Benz(a)anthracene | 100 μg | 70 | 46 | 28 | 18 |
| C | Benz(a)anthracene | 50 μg | 83 | 58 | 32 | 26 |
| D | Benz(a)anthracene 5,6-oxide | 200 μg | 75 | 46 | 22 | 24 |
| E | Benz(a)anthracene 5,6-oxide | 100 μg | 78 | 52 | 27 | 25 |
| F | Benz(a)anthracene 5,6-oxide | 50 μg | 84 | 37 | 18 | 19 |
| G | PEG 400 only | — | 83 | 50 | 26 | 24 |

TABLE II.—*Experiment I: Incidence of Lung, Liver and Other Neoplasms*

| Group | No. of survivors at 50 weeks | 70 weeks | No. of mice examined at p.m. from 50th–70th week with: Liver tumours | Lung tumours | Other tumours | No. of mice examined at p.m. at termination of experiment (70th–75th week) with: Liver tumours | Multiple liver tumours | Lung tumours | Multiple lung tumours | Other tumours |
|---|---|---|---|---|---|---|---|---|---|---|
| A | ♂ 18 | 15 | 1 | 1 | 0 | 15 | 15 | 4 | 4 | 0 |
| | ♀ 18 | 18 | 0 | 0 | 0 | 2 | 1 | 10 | 10 | 1 |
| B | ♂ 25 | 19 | 4 | 1 | 0 | 15 | 11 | 5 | 3 | 1 |
| | ♀ 17 | 13 | 0 | 2 | 0 | 0 | 0 | 5 | 2 | 0 |
| C | ♂ 29 | 25 | 3 | 1 | 0 | 9 | 5 | 5 | 2 | 0 |
| | ♀ 25 | 21 | 0 | 1 | 0 | 2 | 1 | 7 | 3 | 0 |
| D | ♂ 21 | 18 | 2 | 0 | 0 | 5 | 4 | 2 | 1 | 2 |
| | ♀ 21 | 20 | 0 | 0 | 0 | 2 | 0 | 1 | 0 | 0 |
| E | ♂ 27 | 26 | 0 | 1 | 0 | 12 | 8 | 3 | 0 | 0 |
| | ♀ 24 | 22 | 0 | 0 | 0 | 0 | 0 | 6 | 5 | 1 |
| F | ♂ 16 | 16 | 0 | 0 | 0 | 4 | 1 | 5 | 1 | 0 |
| | ♀ 18 | 16 | 0 | 0 | 1 | 0 | 0 | 2 | 0 | 2 |
| G | ♂ 22 | 22 | 0 | 0 | 0 | 4 | 0 | 3 | 1 | 0 |
| | ♀ 23 | 23 | 0 | 0 | 0 | 1 | 0 | 1 | 2 | 0 |

TABLE III.—*Experiment II: Comparison of Effects of Chrysene and 7-Methylbenz(a)-anthracene and their Respective K Region Epoxides and Assessment of the Carcinogenic Activity of the K Region Epoxides of Phenanthrene, Dibenz(a,h)anthracene and 3-Methylcholanthrene*

| Group | Treatment on Day 0 only or on Days 0, 1 and 2 (all treatments given in 0·02 ml PEG 400): Compound | Dose per treatment (μg) | No. of treatments | No. of mice injected (♂ + ♀) | No. of mice weaned (Day 21): Total | ♂ | ♀ |
|---|---|---|---|---|---|---|---|
| H | Chrysene | 100 | 3 | 104 | 51 | 29 | 22 |
| I | Chrysene 5,6-oxide | 100 | 3 | 107 | 47 | 24 | 23 |
| J | 7-Methylbenz(a)anthracene | 100 | 3 | 91 | 49 | 26 | 23 |
| K | 7-Methylbenz(a)anthracene 5,6-oxide | 100 | 3 | 122 | 45 | 20 | 25 |
| L | Phenanthrene 9,10-oxide | 100 | 3 | 111 | 32 | 19 | 13 |
| M | Dibenz(a,h)anthracene 5,6-oxide | 60 | 1 | 93 | 43 | 21 | 22 |
| N | 3-Methylcholanthrene 11,12-oxide | 60 | 1 | 86 | 60 | 31 | 29 |
| O | PEG 400 only | — | 3 | 96 | 52 | 34 | 18 |
| P | PEG 400 only | — | 1 | 94 | 48 | 25 | 23 |

TABLE IV.—*Experiment II: Incidence of Lung, Liver and Other Neoplasms*

| | No. of survivors at | | No. of mice examined at p.m. from 50–70th week with | | | No. of mice examined at p.m. at termination of experiment (70–75th week) with | | | | |
|---|---|---|---|---|---|---|---|---|---|---|
| Group | 50 weeks | 70 weeks | Liver tumours | Lung tumours | Other tumours | Liver tumours | Multiple liver tumours | Lung tumours | Multiple lung tumours | Other tumours |
| H | ♂ 28 | 27 | 0 | 0 | 0 | 13 | 6 | 1 | 1 | 0 |
| | ♀ 22 | 21 | 0 | 0 | 0 | 0 | 0 | 1 | 0 | 0 |
| I | ♂ 23 | 21 | 0 | 0 | 0 | 8 | 3 | 0 | 0 | 0 |
| | ♀ 22 | 20 | 0 | 0 | 0 | 1 | 0 | 0 | 0 | 0 |
| J | ♂ 23 | 4 | 11 | 10 | 0 | 1 | 1 | 3 | 3 | 1 |
| | ♀ 21 | 10 | 0 | 7 | 1 | 0 | 0 | 1 | 1 | 1 |
| K | ♂ 20 | 20 | 0 | 0 | 0 | 4 | 2 | 2 | 2 | 0 |
| | ♀ 25 | 22 | 0 | 1 | 0 | 0 | 0 | 6 | 3 | 0 |
| L | ♂ 19 | 17 | 0 | 0 | 0 | 4 | 1 | 2 | 1 | 0 |
| | ♀ 13 | 13 | 0 | 0 | 0 | 2 | 1 | 0 | 0 | 0 |
| M | ♂ 19 | 16 | 0 | 0 | 0 | 2 | 1 | 0 | 0 | 0 |
| | ♀ 20 | 19 | 0 | 0 | 0 | 0 | 0 | 1 | 0 | 1 |
| N | ♂ 28 | 26 | 0 | 0 | 0 | 6 | 1 | 5 | 0 | 0 |
| | ♀ 27 | 25 | 0 | 0 | 0 | 0 | 0 | 3 | 1 | 0 |
| O | ♂ 33 | 30 | 0 | 0 | 0 | 9 | 1 | 3 | 1 | 0 |
| | ♀ 15 | 15 | 0 | 0 | 0 | 0 | 0 | 1 | 0 | 0 |
| P | ♂ 24 | 20 | 0 | 0 | 0 | 3 | 2 | 1 | 1 | 0 |
| | ♀ 23 | 21 | 0 | 0 | 0 | 3 | 0 | 2 | 1 | 0 |

liver cell masses that were difficult to distinguish from normal liver tissues to pleomorphic, invasive and metastasizing tumours. All the tumours shown in Table II were clearly visible at autopsy and many were over 1 cm in diameter. The lung tumours were all of adenomatous structure, arising in bronchiolar or alveolar epithelium. Most were benign or locally invasive but some had metasstasized within the lobe or origin, or to other lobes of the lung. The lesions shown in Table II ranged in size from 1 mm to 8 mm diameter. Where treatment was associated with an excess of either liver tumours or lung tumours, more tumours of all grades of malignancy were seen. Treatment did not appear to increase the average malignancy of the tumours. A low incidence of other neoplasms was encountered. Most were cases of lymphoma or lymphosarcoma. Their occurrence was not related to treatment.

It is concluded that the epoxide of benz(a)anthracene is less active than its parent compound in increasing liver and lung tumour incidence after parenteral administration during early neonatal life.

*Comparison of chrysene and its K region epoxide*

In Experiment II, the incidence of liver tumours in control male mice given 3 doses of PEG 400 at birth (Group 0) was 9 of 30 animals killed between 70 and 75 weeks. Of these, one had 2 liver tumours. This incidence is higher than in similarly treated mice (Group G) in Experiment I (4 of 22 male mice killed between 70 and 75 weeks, none with multiple liver tumours). Against this background incidence, however, chrysene (Group H) increased liver tumour incidence. Neither chrysene nor its K region epoxide affected the incidence of lung tumours and, if anything, the parent compound seemed to be more active than its epoxide.

*Comparison of 7-methylbenz(a)anthracene and its K region epoxide*

In this case a striking difference was seen between the effects of the 2 compounds on liver tumour incidence in males. Most of the animals treated with the parent compound died, or had to be killed because they were sick, between the 50th and 70th week of the experiment. Nevertheless, a much higher incidence of liver tumours was encountered in these animals than in animals treated with the epoxide that were examined post mortem between the 70th and 75th week. The females of both groups exhibited a raised incidence of lung tumours and in this case the activity of the epoxide was not less than that of the parent compound.

*K region epoxides of phenanthrene, dibenz-(a,h)anthracene and 3-methylcholanthrene*

In the doses tested, all 3 epoxides were without obvious effect on the incidence of liver, lung or other tumours. It is highly probable that the epoxides of dibenz(a,h)anthracene and 3-methylcholanthrene are less active than the parent compounds and that the epoxide of phenanthrene is as active as phenanthrene itself. Groups of mice treated with the 3 parent compounds were not included, however, in the present experiment.

### DISCUSSION

The carcinogenic activity of some K region epoxides derived from polycyclic hydrocarbons has been tested in newborn mice partly because of the conflicting results that have previously been obtained from tests in adult animals and from *in vitro* malignant transformation systems. There are obvious difficulties associated with testing reactive intermediates like the hydrocarbon epoxides for carcinogenicity and it was thought that rapidly dividing neonatal tissues might offer some advantages over those of adult animals, especially if factors such as reactions with DNA at a critical stage of the cell cycle, DNA replication and DNA repair are involved in carcinogenesis. Additionally, neonatal tissues probably possess lower levels of the microsomal epoxide hydrase that catalyses the conversion of epoxides to dihydrodiols (Oesch, 1973).

The results obtained in the present experiments have confirmed the activity of the hydrocarbons benz(a)anthracene, 7-methylbenz(a)anthracene and chrysene in newborn mice, but show that the K region epoxides related to these 3 hydrocarbons were not more active as carcinogens than the parent hydrocarbons in this test system. Although direct comparisons were not carried out here, the results also indicate that the K region epoxides derived from dibenz(a,h)anthracene and 3-methylcholanthrene are likely to be less active than the corresponding hydrocarbons, which have previously been tested in newborn mice (Kelly and O'Gara, 1961). The phenanthrene epoxide did not appear to enhance tumour incidence, which is not really surprising since the parent hydrocarbon is also inactive in newborn mice (Roe and Walters, 1967).

In the metabolism of the polycyclic hydrocarbons, a variety of epoxides seem certain to be formed as the initial products of the oxidation of the aromatic double bonds, a process catalysed by the microsomal mono-oxygenases, and it is these epoxides that are now thought to be converted into the hydroxylated derivatives that are the main metabolites of the hydrocarbons. So far, most studies of hydrocarbon epoxides have been concentrated, for practical reasons, on K region epoxides, several of which have been detected as metabolites and prepared by synthesis, and this work has recently been reviewed (Sims and Grover, 1974). Although some K region epoxides were more active than the parent hydrocarbons in the induction of malignant transformation of cells in culture, most K region epoxides were found to be less carcinogenic in adult animals and, as reported here, are less active in newborn mice. In addition, in cells treated with 7-methylbenz(a)anthracene, the DNA products formed are different from those obtained with the corresponding K region epoxide (Baird *et al.*, 1973). On balance therefore, these findings can be interpreted as meaning that K region epoxides are not the epoxide metabolites involved in hydrocarbon carcinogenesis. This is in accord with some very recent evidence which suggests that the DNA products that are formed in mammalian cells treated with polycyclic hydrocarbons arise from non-K region epoxides, which are themselves derived from dihydrodiols (Sims *et al.*, 1974; Swaisland *et al.*, 1974); this new type of diol-epoxide has not yet been examined for biological activity.

We wish to thank Norma Barron for valuable technical assistance. This research was supported by grants to the Chester Beatty Research Institute: Institute of Cancer Research, Royal Cancer Hospital from the Cancer Research Campaign and the Medical Research Council.

### REFERENCES

AMES, B. N., SIMS, P. & GROVER, P. L. (1972) Epoxides of Carcinogenic Polycyclic Hydrocarbons as Frameshift Mutagens. *Science, N.Y.*, **176**, 47.

BAIRD, W. M., DIPPLE, A., GROVER, P. L., SIMS, P. & BROOKES, P. (1973) Studies on the Formation of Hydrocarbon-Deoxyribonucleoside Products by the Binding of Derivatives of 7-Methylbenz(a)-anthracene to DNA in Aqueous Solution and in Mouse Embryo Cells in Culture. *Cancer Res.*, **33**, 2386.

BERTRAM, J. S. & HEIDELBERGER, C. (1974) Cell Cycle Dependency of Oncogenic Transformation Induced by N-Methyl-N′-nitro-N-nitrosoguanidine in Culture. *Cancer Res.*, **34**, 526.

BERWALD, Y. & SACHS, L. (1963) *In vitro* Cell Transformation with Chemical Carcinogens. *Nature, Lond.*, **200**, 1182.

BOYLAND, E. & SIMS, P. (1965) The Metabolism of Benz(a)anthracene and Dibenz(a,h)anthracene and their 5,6-Epoxy-5,6-dihydro Derivatives by Rat Liver Homogenates. *Biochem. J.*, **97**, 7.

BOYLAND, E. & SIMS, P. (1967) The Carcinogenic Activities in Mice of Compounds Related to Benz(a)anthracene. *Int. J. Cancer*, **2**, 500.

CHEN, T. T. & HEIDELBERGER, C. (1969) Cultivation *in vitro* of Cells Derived from Adult C3H Mouse Ventral Prostate. *J. natn. Cancer Inst.*, **42**, 903.

COOKSON, M. J., SIMS, P. & GROVER, P. L. (1971) Mutagenicity of Epoxides of Polycyclic Hydrocarbons Correlates with Carcinogenicity of Parent Hydrocarbon. *Nature, New Biol.*, **234**, 186.

FAHMY, O. G. & FAHMY, M. J. (1973) Oxidative Activation of Benz(a)anthracene and Methylated Derivatives in Mutagenesis and Carcinogenesis. *Cancer Res.*, **33**, 2354.

GROVER, P. L. & SIMS, P. (1970) Interactions of the K Region Epoxides of Phenanthrene and Dibenz(a,h)anthracene with Nucleic Acids and Histone. *Biochem. Pharmac.*, **19**, 2251.

GROVER, P. L., HEWER, A. & SIMS, P. (1971) Epoxides as Microsomal Metabolites of Polycyclic Hydrocarbons. *FEBS Letters*, **18**, 76.

GROVER, P. L., HEWER, A. & SIMS, P. (1972) The Formation of Epoxides as Microsomal Metabolites of Pyrene and Benzo(a)pyrene. *Biochem. Pharmac.*, **21**, 2713.

GROVER, P. L., SIMS, P., HUBERMAN, E., MARQUARDT, H., KUROKI, T. & HEIDELBERGER, C. (1971) *In vitro* Transformation of Rodent Cells by K-Region Derivatives of Polycyclic Hydrocarbons. *Proc. natn. Acad. Sci. U.S.A.*, **68**, 1098.

HUBERMAN, E., ASPIRAS, L., HEIDELBERGER, C., GROVER, P. L. & SIMS, P. (1971) Mutagenicity to Mammalian Cells of Epoxides and Other Derivatives of Polycyclic Hydrocarbons. *Proc. natn. Acad. Sci. U.S.A.*, **68**, 3195.

HUBERMAN, E., KUROKI, T., MARQUARDT, H., SELKIRK, J. K., HEIDELBERGER, C., GROVER, P. L. & SIMS, P. (1972) Transformation of Hamster Embryo Cells by Epoxides and Other Derivatives of Polycyclic Hydrocarbons. *Cancer Res.*, **32**, 1391.

KELLY, M. G. & O'GARA, R. W. (1961) Induction of Tumors in Newborn Mice with Dibenz(a,h)-anthracene and 3-Methylcholanthrene. *J. natn. Cancer Inst.*, **26**, 651.

KEYSELL, G. R., BOOTH, J., GROVER, P. L., HEWER, A. & SIMS, P. (1973) The Formation of K-Region Epoxides as Hepatic Microsomal Metabolites of 7-Methylbenz(a)anthracene and 7,12-Dimethylbenz(a)anthracene and their 7-Hydroxymethyl Derivatives. *Biochem. Pharmac.*, **22**, 2853.

MARQUARDT, H. (1974) Cell-cycle Dependence of Chemically Induced Malignant Transformation *in vitro*. *Cancer Res.*, **34**, 1612.

MARQUARDT, H., KUROKI, T., HUBERMAN, E., SELKIRK, J. K., HEIDELBERGER, C., GROVER, P. L. & SIMS, P. (1972) Malignant Transformation of Cells Derived from Mouse Prostate by Epoxides and Other Derivatives of Polycyclic Hydrocarbons. *Cancer Res.*, **32**, 716.

MARQUARDT, H., SODERGREN, J. E., SIMS, P. & GROVER, P. L. (1974) Malignant Transformation *in vitro* of Mouse Fibroblasts by 7,12-Dimethylbenz(a)anthracene and 7-Hydroxymethylbenz-(a)anthracene and by Their K-Region Derivatives. *Int. J. Cancer*, **13**, 304.

MILLER, E. C. & MILLER, J. A. (1967) Low Carcinogenicity of the K-Region Epoxides of 7-Methylbenz(a)anthracene and Benz(a)anthracene in the Mouse and Rat. *Proc. Soc. exp. Biol. Med.*, **124**, 915.

NEWMAN, M. S. & BLUM, S. (1964) A New Cyclization Reaction Leading to Epoxides of Aromatic Hydrocarbons. *J. Am. chem. Soc.*, **86**, 5598.

OESCH, F. (1973) Mammalian Epoxide Hydrases: Inducible Enzymes Catalysing the Inactivation of Carcinogenic and Cytotoxic Metabolites Derived from Aromatic and Olefinic Compounds. *Xenobiotica*, **3**, 305.

ROE, F. J. C. & WALTERS, M. A. (1967) Induction of Hepatoma in Mice by Carcinogens of the Polycyclic Hydrocarbon Type. *Nature, Lond.*, **214**, 299.

ROE, F. J. C., MITCHLEY, B. C. V. & WALTERS, M. (1963) Tests for Carcinogenesis Using Newborn

Mice; 1,2-Benzanthracene,2-Naphthylamine, 2-Naphthylhydroxylamine and Ethyl Methane Sulphonate. *Br. J. Cancer*, **17**, 255.

ROE, F. J. C., ROWSON, K. E. K. & SALAMAN M. H. (1961) Tumours of Many Sites Induced by Injection of Chemical Carcinogens into Newborn Mice, A Sensitive Test for Carcinogenesis: The Implications for Certain Immunological Theories. *Br. J. Cancer*, **15**, 515.

SIMS, P. (1966) The Metabolism of 3-Methylcholanthrene and Some Related Compounds by Rat-liver Homogenates. *Biochem. J.*, **98**, 215.

SIMS, P. (1967) The Carcinogenic Activities in Mice of Compounds Related to 3-Methylcholanthrene. *Int. J. Cancer*, **2**, 505.

SIMS, P. & GROVER, P. L. (1974) Epoxides in Polycyclic Aromatic Hydrocarbon Metabolism and Carcinogenesis. *Adv. Cancer Res.*, **20**, 165.

SIMS, P., GROVER, P. L., SWAISLAND, A. J., PAL, K. & HEWER, A. (1974) Metabolic Activation of Benzo(a)pyrene Proceeds *via* a Diol Epoxide. *Nature, Lond.* In the press.

SWAISLAND, A. J., HEWER, A., PAL, K., KEYSELL, G. R., BOOTH, J., GROVER, P. L. & SIMS, P. (1974) Polycyclic Hydrocarbon Epoxides: The Involvement of 8,9-Dihydro-8,9-dihydroxybenz-(a)anthracene 10,11-oxide in Reactions with the DNA of Benz(a)anthracene Treated Hamster Embryo Cells. *FEBS Letters.*, **47**, 34.

VAN DUUREN, B. L., LANGSETH, L., GOLDSCHMIDT, B. M. & ORRIS, L. (1967) Carcinogenicity of Epoxides, Lactones and Peroxy Compounds. VI. Structure and Carcinogenic Activity. *J. natn. Cancer Inst.*, **39**, 1217.

WALTERS, M. A. & ROE, F. J. C. (1964) The Effect of Dietary Casein on the Induction of Lung Tumours by the Injection of 9,10-Dimethyl-1,2-benzanthracene (DMBA) into Newborn Mice. *Br. J. Cancer*, **18**, 312.

WALTERS, M. A. & ROE, F. J. C. (1966) The Time of Appearance of Lung Tumours in Mice Injected when Newly Born with 9,10-Dimethyl-1,2-benzanthracene (DMBA). *Br. J. Cancer*, **20**, 161.


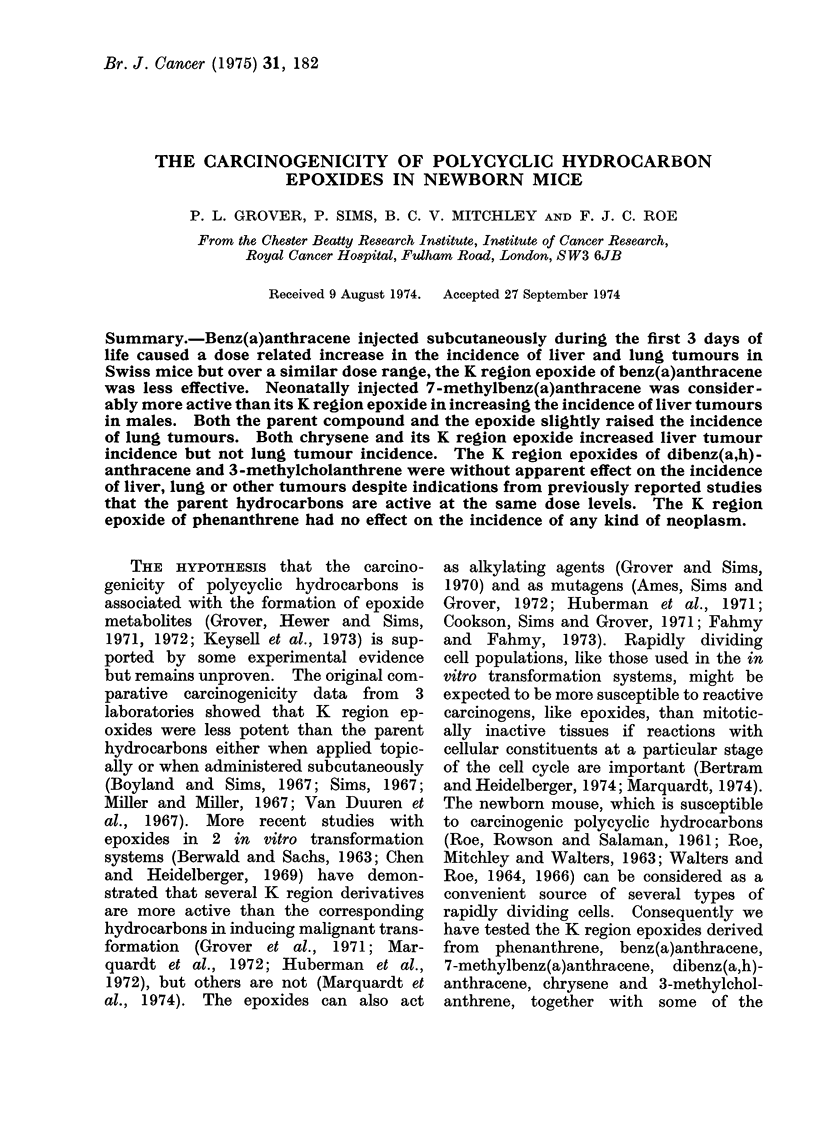

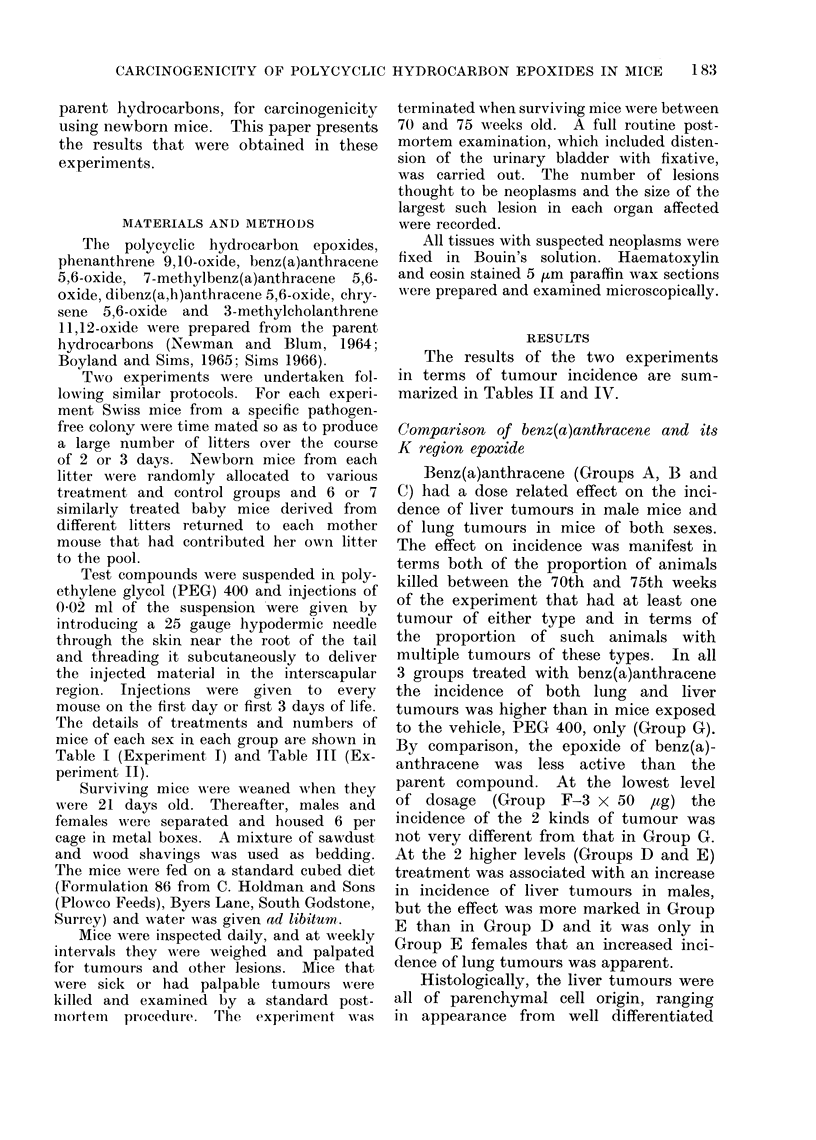

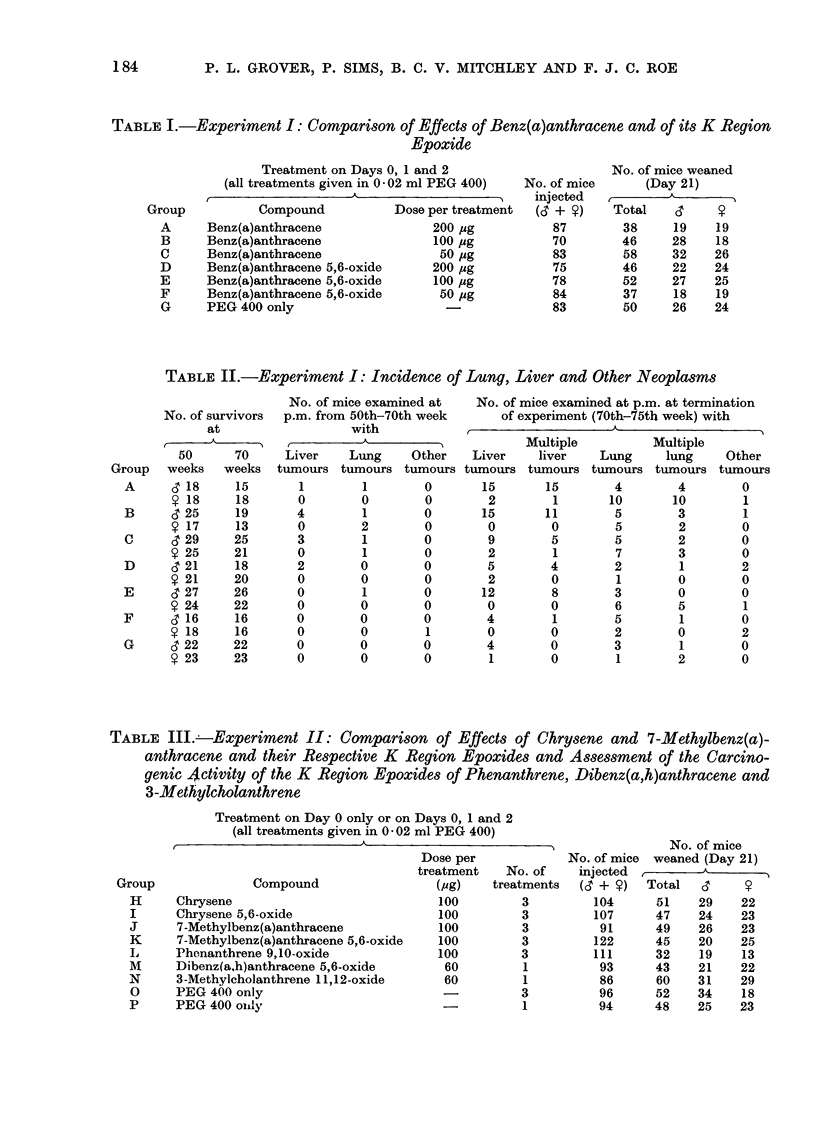

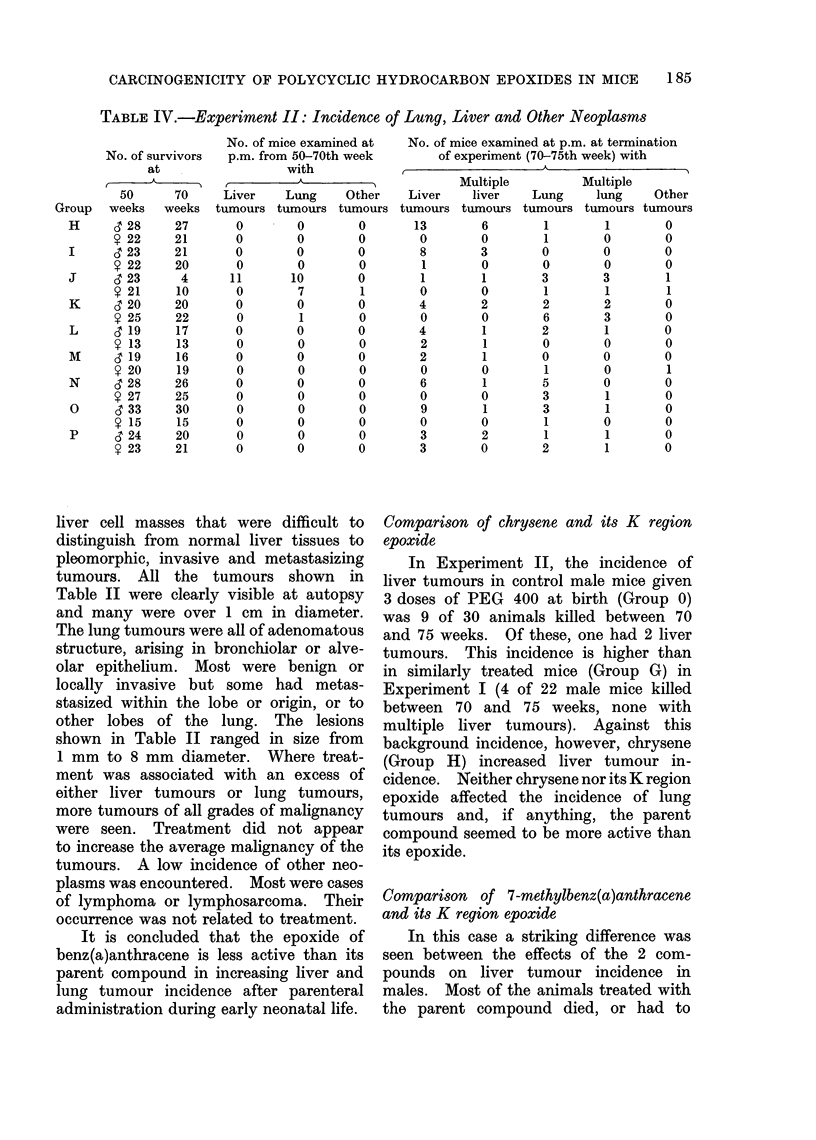

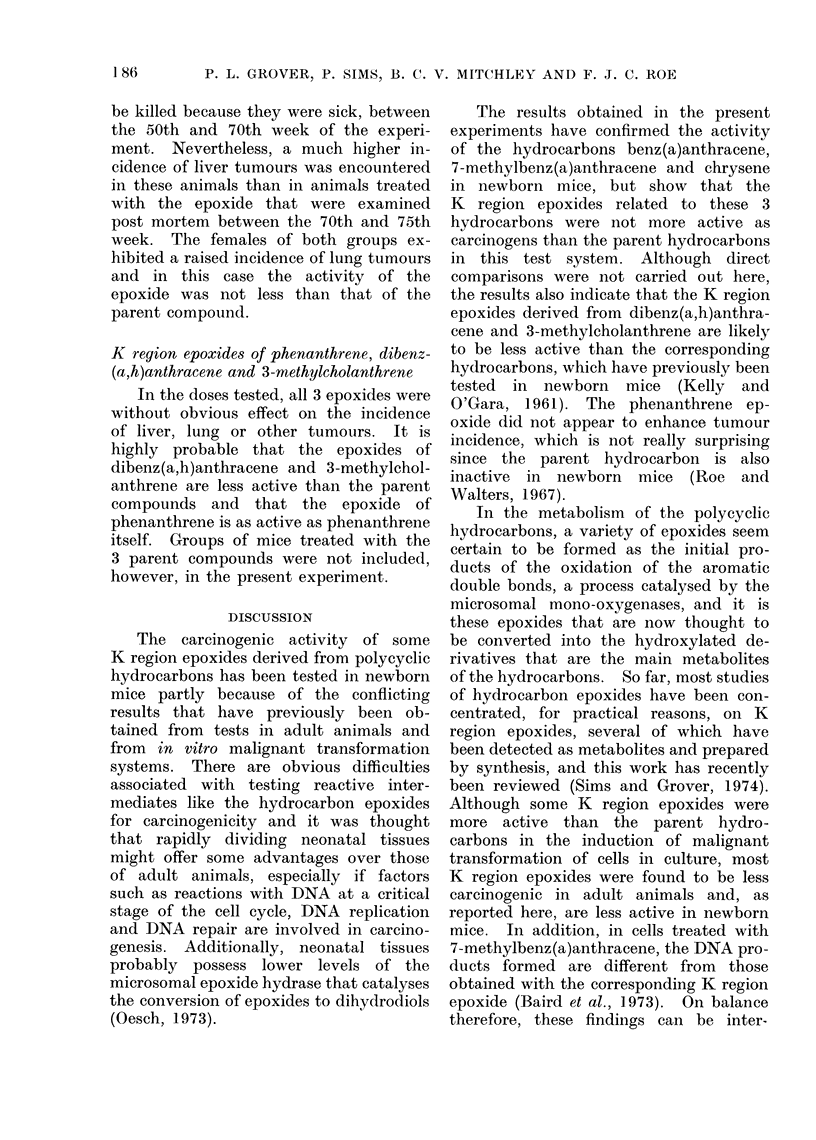

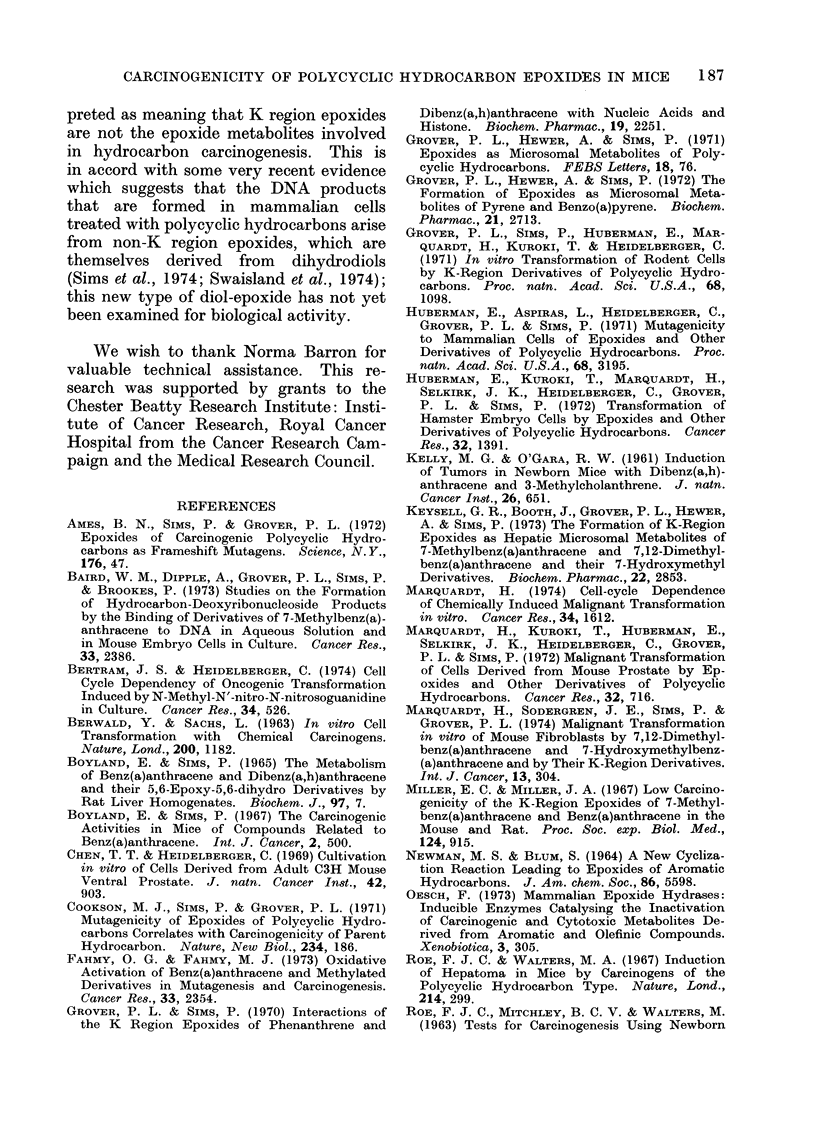

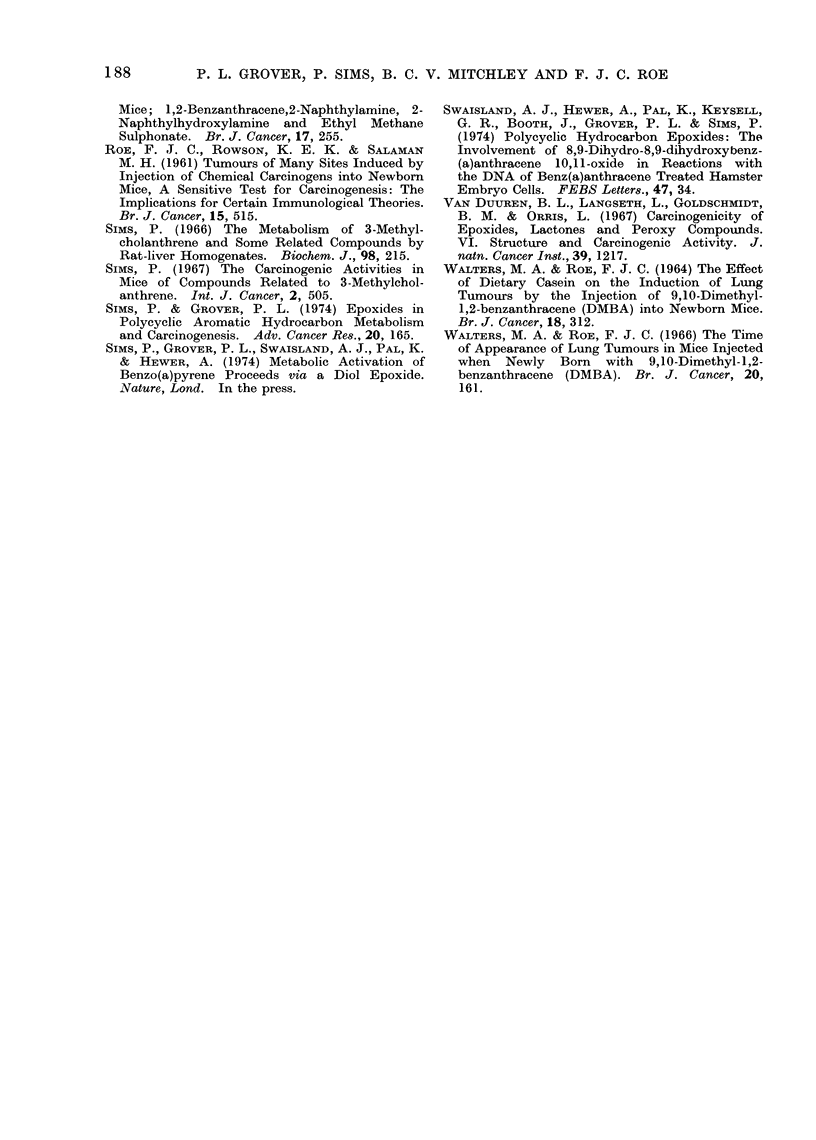


## References

[OCR_00900] Ames B. N., Sims P., Grover P. L. (1972). Epoxides of carcinogenic polycyclic hydrocarbons are frameshift mutagens.. Science.

[OCR_00921] BERWALD Y., SACHS L. (1963). IN VITRO CELL TRANSFORMATION WITH CHEMICAL CARCINOGENS.. Nature.

[OCR_00906] Baird W. M., Dipple A., Grover P. L., Sims P., Brookes P. (1973). Studies on the formation of hydrocarbon-deoxyribonucleoside products by the binding of derivatives of 7-methylbenz(a)anthracene to DNA in aqueous solution and in mouse embryo cells in culture.. Cancer Res.

[OCR_00915] Bertram J. S., Heidelberger C. (1974). Cell cycle dependency of oncogenic transformation induced by N-methyl-N'-nitro-N-nitrosoquanidine in culture.. Cancer Res.

[OCR_00932] Boyland E., Sims P. (1967). The carcinogenic activities in mice of compounds related to benz[a]anthracene.. Int J Cancer.

[OCR_00926] Boyland E., Sims P. (1965). The metabolism of benz[a]anthracene and dibenz[a,h]anthracene and their 5,6-epoxy-5,6-dihydro derivatives by rat-liver homogenates.. Biochem J.

[OCR_00937] Chen T. T., Heidelberger C. (1969). Cultivation in vitro of cells derived from adult C3H mouse ventral prostate.. J Natl Cancer Inst.

[OCR_00943] Cookson M. J., Sims P., Grover P. L. (1971). Mutagenicity of epoxides of polycyclic hydrocarbons correlates with carcinogenicity of parent hydrocarbons.. Nat New Biol.

[OCR_00949] Fahmy O. G., Fahmy M. J. (1973). Oxidative activation of benz(a)anthracene and methylated derivatives in mutagenesis and carcinogenesis.. Cancer Res.

[OCR_00962] Grover P. L., Hewer A., Sims P. (1971). Epoxides as microsomal metabolites of polycyclic hydrocarbons.. FEBS Lett.

[OCR_00967] Grover P. L., Hewer A., Sims P. (1972). Formation of K-region epoxides as microsomal metabolites of pyrene and benzo(a)pyrene.. Biochem Pharmacol.

[OCR_00975] Grover P. L., Sims P., Huberman E., Marquardt H., Kuroki T., Heidelberger C. (1971). In vitro transformation of rodent cells by K-region derivatives of polycyclic hydrocarbons.. Proc Natl Acad Sci U S A.

[OCR_00955] Grover P. L., Sims P. (1970). Interactions of the K-region epoxides of phenanthrene and dibenz (a,h)anthracene with nucleic acids and histone.. Biochem Pharmacol.

[OCR_00981] Huberman E., Aspiras L., Heidelberger C., Grover P. L., Sims P. (1971). Mutagenicity to mammalian cells of epoxides and other derivatives of polycyclic hydrocarbons.. Proc Natl Acad Sci U S A.

[OCR_00988] Huberman E., Kuroki T., Marquardt H., Selkirk J. K., Heidelberger C., Grover P. L., Sims P. (1972). Transformation of hamster embryo cells by epoxides and other derivatives of polycyclic hydrocarbons.. Cancer Res.

[OCR_00996] KELLY M. G., O'GARA R. W. (1961). Induction of tumors in newborn mice with dibenz[a, h]anthracene and 3-methylcholanthrene.. J Natl Cancer Inst.

[OCR_01002] Keysell G. R., Booth J., Grover P. L., Hewer A., Sims P. (1973). The formation of "K-region" epoxides as hepatic microsomal metabolites of 7-methylbenz(a)anthracene and 7,12-dimethylbenz(a)anthracene and their 7-hydroxymethyl derivatives.. Biochem Pharmacol.

[OCR_01010] Marquardt H. (1974). Cell cycle dependence of chemically induced malignant transformation in vitro.. Cancer Res.

[OCR_01015] Marquardt H., Kuroki T., Huberman E., Selkirk J. K., Heidelberger C., Grover P. L., Sims P. (1972). Malignant transformation of cells derived from mouse prostate by epoxides and other derivatives of polycyclic hydrocarbons.. Cancer Res.

[OCR_01023] Marquardt H., Sodergren J. E., Sims P., Grover P. L. (1974). Malignant transformation in vitro of mouse fibroblasts by 7,12-dimethylbenz(A)anthracene and 7-hydroxymethylbenz(A)anthracene and by their K-region derivatives.. Int J Cancer.

[OCR_01031] Miller E. C., Miller J. A. (1967). Low carcinogenicity of the K-region epoxides of 7-methylbenz(a)-anthracene and benz(a)anthracene in the mouse and rat.. Proc Soc Exp Biol Med.

[OCR_01043] Oesch F. (1973). Mammalian epoxide hydrases: inducible enzymes catalysing the inactivation of carcinogenic and cytotoxic metabolites derived from aromatic and olefinic compounds.. Xenobiotica.

[OCR_01056] ROE F. J., MITCHLEY B. C., WALTERS M. (1963). TESTS FOR CARCINOGENESIS USING NEWBORN MICE: 1,2BENZANTHRACENE, 2-NAPHTHYLAMINE, 2-NAPHTHYLHYDROXYLAMINE AND ETHYL METHANE SULPHONATE.. Br J Cancer.

[OCR_01066] ROE F. J., ROWSON K. E., SALAMAN M. H. (1961). Tumours of many sites induced by injection of chemical carcinogens into newborn mice, a sensitive test for carcinogenesis: the implications for certain immunological theories.. Br J Cancer.

[OCR_01050] Roe F. J., Waters M. A. (1967). Induction of hepatoma in mice by carcinogens of the polycyclic hydrocarbon type.. Nature.

[OCR_01084] Sims P., Grover P. L. (1974). Epoxides in polycyclic aromatic hydrocarbon metabolism and carcinogenesis.. Adv Cancer Res.

[OCR_01079] Sims P. (1967). The carcinogenic activities in mice of compounds related to 3-methylcholanthrene.. Int J Cancer.

[OCR_01074] Sims P. (1966). The metabolism of 3-methylcholanthrene and some related compounds by rat-liver homogenates.. Biochem J.

[OCR_01095] Swaisland A. J., Hewer A., Pal K., Keysell G. R., Booth J., Grover P. L., Sims P. (1974). Polycyclic hydrocarbon epoxides: the involvement of 8,9-dihydro-8,9-dihydroxybenz (a) anthracene 10,11-oxide in reactions with the DNA of benz (a) anthracene-treated hamster embryo cells.. FEBS Lett.

[OCR_01104] Van Duuren B. L., Langseth L., Goldschmidt B. M., Orris L. (1967). Carcnogenicity of epoxides, lactones, and peroxy compounds. VI. Structure and carcinogenic activity.. J Natl Cancer Inst.

[OCR_01111] WALTERS M. A., ROE F. J. (1964). THE EFFECT OF DIETARY CASEIN ON THE INDUCTION OF LUNG TUMOURS BY THE INJECTION OF 9,10-DIMETHYL-1,2-BENZANTHRACENE (DMBA) INTO NEWBORN MICE.. Br J Cancer.

[OCR_01118] Walters M. A., Roe F. J. (1966). The time of appearance of lung tumours in mice injected when newly born with 9,10-dimethyl-1,2-benzathracene (DMBA).. Br J Cancer.

